# Human *MLL/KMT2A* gene exhibits a second breakpoint cluster region for recurrent MLL–USP2 fusions

**DOI:** 10.1038/s41375-019-0451-7

**Published:** 2019-03-21

**Authors:** Claus Meyer, Bruno A. Lopes, Aurélie Caye-Eude, Hélène Cavé, Chloé Arfeuille, Wendy Cuccuini, Rosemary Sutton, Nicola C. Venn, Seung Hwan Oh, Grigory Tsaur, Gabriele Escherich, Tobias Feuchtinger, Hansen J. Kosasih, Seong L. Khaw, Paul G. Ekert, Maria S. Pombo-de-Oliveira, Audrey Bidet, Bardya Djahanschiri, Ingo Ebersberger, Marketa Zaliova, Jan Zuna, Zuzana Zermanova, Vesa Juvonen, Renate Panzer Grümayer, Grazia Fazio, Gianni Cazzaniga, Patrizia Larghero, Mariana Emerenciano, Rolf Marschalek

**Affiliations:** 1 DCAL/Institute of Pharmaceutical Biology, Goethe-University Frankfurt, Frankfurt am Main, Germany; 2 Division of Clinical Research, Research Center, Instituto Nacional de Cancer, Rio de Janeiro, Brazil; 3 Department of Genetics, AP-HP Robert Debré, Paris Diderot University, Paris, France; 4 Department of Cytogenetics, Saint Louis Hospital, Paris, France; 5 Children’s Cancer Institute Australia, University of NSW Sydney, Sydney, Australia; 6 Department of Laboratory Medicine, Inje University College of Medicine, Busan, Korea; 7 Regional Children Hospital 1, Research Institute of Medical Cell Technologies, Pediatric Oncology and Hematology Center, Ural Federal University named after the first President of Russia BN Yeltsin, Ekaterinburg, Russia; 8 Clinic of Pediatric Hematology and Oncology, University Medical Center Hamburg-Eppendorf, Hamburg, Germany; 9 Department of Pediatric Hematology, Oncology, Hemostaseology and Stem Cell Transplantation, Dr. von Hauner University Children’s Hospital, Ludwig Maximilian University Munich, Munich, Germany; 10 Murdoch Children’s Research Institute, The Royal Children’s Hospital, Flemington Road Parkville, 3052 Victoria, Australia; 11 Pediatric Hematology-Oncology Program—Research Center, Instituto Nacional de Cancer Rio de Janeiro, Rio de Janeiro, Brazil; 12 Laboratoire d’Hématologie, University Hospital, Brest, CHU de Bordeaux, Bordeaux, France; 13 Department of Applied Bioinformatics, Institute of Cell Biology and Neuroscience, Goethe-Universität Frankfurt, Frankfurt am Main, 60438 Germany; 14 Senckenberg Climate and Research Centre (BIK-F), Frankfurt am Main, 60325 Germany; 15 CLIP, Department of Paediatric Haematology/Oncology, Charles University Prague, 2nd Faculty of Medicine, Prague, Czech Republic; 16 Center of Oncocytogenetics, Institute of Medical Biochemistry and Laboratory Diagnostics, General University Hospital and First Faculty of Medicine, Charles University, Prague, Czech Republic; 17 Department of Clinical Chemistry and Laboratory Division, Turku University Hospital, Turku, Finland; 18 Children’s Cancer Research Institute, Medical University of Vienna, Vienna, Austria; 19 Centro Ricerca Tettamanti, Clinica Pediatrica University of Milano-Bicocca, Monza, Italy

**Keywords:** Genetics research, Acute lymphocytic leukaemia

**To the Editor,**

For nearly 3 decades, the human *MLL (KMT2A)* gene and its rearrangements have been investigated in many different laboratories around the world. At our diagnostic center (DCAL Frankfurt), our standard strategy for the identification of *MLL*-r is based on two independent approaches, namely “Multiplex” (MP)-polymerase chain reaction (PCR) and “Long distance inverse” (LDI)-PCR approach [1]. The MP-PCR approach is used to rapidly identify the eight most frequent *MLL* fusions (*AF4*, *AF6*, *AF9*, *AF10*, *ENL*, *ELL*, *EPS15*, and *PTDs*) which encompass ~90% of all diagnosed *MLL*-r leukemia patients, while LDI-PCR is used for all other patients (~10%). By applying both technologies, we have accumulated 94 direct *MLL-*gene fusions and 247 reciprocal fusion partner genes [2]. Nearly, all breakpoints have been identified in the major breakpoint cluster region (BCR) of the *MLL* gene (*MLL* exons 8–14). However, some of the patients remained negative, although they were positively prescreened by various methods.

In order to diagnose *MLL* breakpoints in every patient, a total of 2688 overlapping Illumina capture probes covering the whole-*MLL* gene were designed and used to analyze a cohort of AL patients (*n* = 109) where we had either limited (*n* = 4; PCR positive but not sequenced) or no information (*n* = 105) on their molecular status. As depicted in Fig. 1a, we identified chromosomal rearrangements in 93 out of 109 patient cases. Sixteen patients remained *MLL*-r negative and were therefore assigned as patients with “unknown status”. The data analyses of the remaining 93 patients revealed the following distribution: for 67 patients (72%) a breakpoint could be analyzed in the major BCR; 5 patients (5%) displayed only the reciprocal der(TP) with breakpoints in exon 11 (putative CEP83-MLL spliced fusion), intron 11 (*n* = 3; putative FKBP8-MLL spliced fusion, AF9-MLL, RELA-MLL) and intron 27 (IFT46-MLL), respectively. Surprisingly, an additional 21 patients (23%) had their breakpoints outside of the major BCR, but inside a novel, minor BCR. This novel BCR is localizing between *MLL* intron 19 and exon 24 (with a clear preference for *MLL* intron 21–23).

**Fig. 1:**
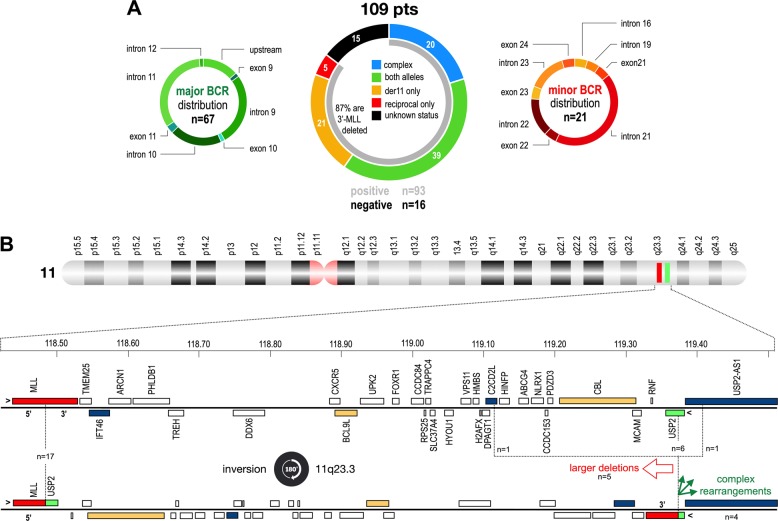
Overview about all analyzed patients, their molecular information and breakpoint distribution. **a** Data from 109 patients which were analyzed with NGS in percentages. Their breakpoint distribution is displayed left (major BCR; *n* = 67) and right (minor BCR; *n* = 21). Five patients displayed only a reciprocal fusion, while 16 cases displayed no *MLL* rearrangement. **b** Top: chromosome 11 is depicted with highlighting of the *MLL* (red) and *USP2* (green) genes. Below: all the genes between *MLL* and *USP*; blue marked genes: additional genes found in this study to be rearranged with *MLL*; orange marked genes: genes that have been earlier described to be rearranged with *MLL*. Recombinations between *MLL* and *USP2* are caused by an inversion, with reciprocal alleles that carry additional deletions or complex rearrangements

Most of the new BCR cases represented *MLL–USP2* gene fusions (*n* = 17). *USP2* is localized about 1 Mbp telomer to *MLL* at 11q23.3 and transcriptionally orientated in direction of the centromere of chromosome 11, classifying all these fusions as intrachromosomal inversions (see Fig. 1b). In addition, we identified four balanced translocations in the minor BCR: one patient with an *USP8* fusion (see Fig. 2 and Suppl. Figure S1), two with *AF4* and one with *AF9*.

**Fig. 2:**
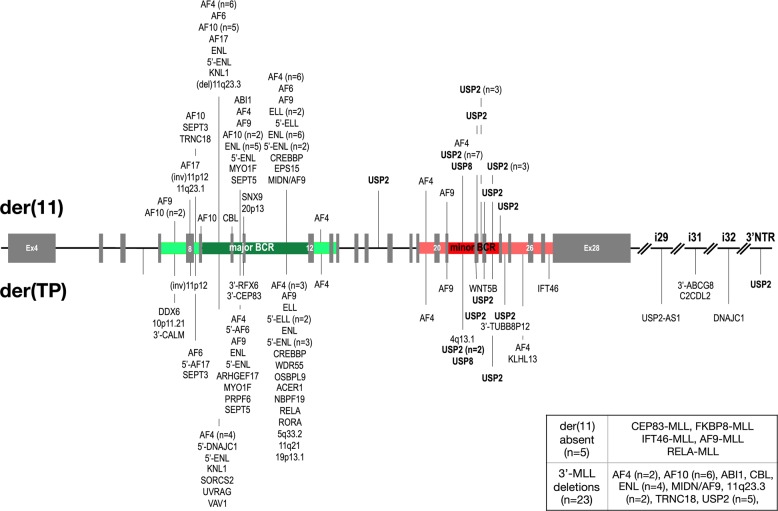
Detailed distribution of all breakpoints in both BCRs of MLL. The *MLL* gene is depicted from exon 4 to the end. The major BCR is marked in green, the minor BCR in red. Main breakpoint regions are depicted in dark green/red while regions with fewer breakpoints are depicted in light green/red. The fusions sites and the fusion partners are shown. Information about the 5 cases with no der(11) or the 23 cases with 3′-MLL deletions are given in the box at the right bottom

*MLL–USP2 and MLL–USP8* alleles seem to be restricted to the minor BCR (see Fig. 2), because they were never diagnosed in association with the major BCR. Most of the reciprocal *USP2–MLL* fusions were scattered over a larger region at 11q23.3 (see Fig. 2), involving also upstream (C2CD2L) and downstream genes (USP2-AS1). Our analysis revealed also five patients with 3′-*MLL* deletions that were caused by microdeletions (<200 bp), larger deletions (up to 34 kbp), or complex rearrangements including other chromosomes as well (*n* = 4; chromosome regions 2p21, 4q13.1, 12p13.33, and 18p11.32). A detailed picture of the investigated *MLL–USP2* and *MLL–USP8* and their reciprocal fusions is shown in the Suppl. Fig. S2A–D.

All patients with a rearrangement of *USP2* or *USP8* fused the conserved “UCH-domain” to an extended 5′-MLL portion (see Suppl. Fig. S1A). This may indicate that the UCH domain has a functional importance for the resulting MLL fusion protein. *USP* genes belong to a large group of deubiquitinating proteins binding to specific target proteins [3–5]. The USP family exhibits a ubiquitin-specific protease (UCH domain) that is characterized by several conserved amino acids that are summarized as CYS- and ASP-box (see Suppl. Fig. 1B). USP2 protein deubiquinates and stabilizes MDM2, leading to an enhanced degradation of p53 [6]. This in turn activates MYC, because active p53 induces the transcription of several microRNAs that target MYC mRNA.

MLL fusions with the conserved 3′-UCH domain of USP2 and USP8 may change profoundly the functions of these novel MLL fusion proteins. It has already been shown that PHD2 [7] and PHD3/BD [8] both bind to proteins (CDC34 and ESC^ASB2^) that mediate the destruction of MLL by poly-ubiquitination and proteasomal degradation. Fusing single or all PHD domains to a der(11) product (MLL-AF9 and MLL-ENL) caused even a strong drop of their transforming potential [9, 10]. This well-described degradation mechanism of MLL may now be counteracted by the UCH domain of MLL–USP2 or MLL–USP8, and thus, restoring their oncogenic transformation capacity.

In our cohort, we also identified new *MLL* fusion partner genes (*n* = 3). These novel fusion genes were *SNX9* (6q25.3), *USP8* (15q21.2), and *SEPT3* (22q13.2). *SNX9* encodes a protein known to be a member of the sorting nexin family which contain a phosphoinositide binding domain and are involved in intracellular trafficking. The SNX9 protein has a variety of interaction partners, including an adapter protein 2, dynamin, tyrosine kinase nonreceptor 2, Wiskott–Aldrich syndrome-like, and ARP3 actin-related protein 3. *USP8* has diverse functions, being required for the internalization of liganded receptor tyrosine kinases and stabilization of ESCRT components. The USP8 protein is thought to regulate the morphology of the endosome by ubiquitination of proteins on this organelle and is involved in cargo sorting and membrane trafficking at the early endosome stage. *SEPT3* is the seventh member of the septin family of GTPases that is fused to *MLL*. Members of this family are required for cytokinesis.

A few cases of *MLL–USP2* fusions have already been described. However, these were single patient cases and they were classified as exceptional rearrangements [11–13]. Our NGS approach allowed for the first time the recurrent characterization of breakpoints in this novel minor BCR region of the *MLL* gene. Moreover, our targeted NGS approach enabled us to overcome the technical limitations associated with LDI-PCR and MP-PCR approaches.

Another advantage of the targeted NGS approach is the simultaneous identification of 3′ *MLL* deletions or copy number variations. In the current study, 23 of the patients (out of 88: 26%) had a 3′ *MLL* deletion. According to our data, 3′-*MLL* deletions were present in both breakpoint groups (major and minor) to a similar extent with 26.9% and 23.8%, respectively. This seems to be much higher than previously described (Andersson et al. [12]: 13%; Peterson et al. [14]: 7%).

In diagnostic fluorescence in situ hybridization analyses, these *MLL–USP2* cases revealed two major patterns: (1) loss of the 3′-*MLL* probe signal, and (2) a normal pattern typical for *MLL* wild-type (Suppl. Table S1b, Suppl. Fig. S3). Considering the clinical data (Suppl. Table S1a–c), our 17 patients with *MLL–USP2* were divided into 8 males and 9 females. All of them were children, and the median age at diagnosis was 17 months (range: 3–120 months). The median leukocyte count was 30.4 × 10^9^/L (range: 3.4–324.0 × 10^9^/L), and the disease phenotype was predominantly B-ALL (*n* = 12), followed by mixed-phenotype acute leukemia (MPAL) (*n* = 4) and acute myeloid leukemia (*n* = 1). The MPAL cases all had mixed myeloid and B-cell phenotype. The patients were treated with diverse therapy protocols. Five patients (29%) presented with central nervous system disease, and 13 patients (76%) had positive-minimal residual disease (MRD) levels at day 33. Prednisone response was measured in 12 patients with a poor response in 5 patients (42%). The median follow-up of the patients was 1.2 years (range: 0.1–11.1 years), and 2 cases died after 5 and 9 months following diagnosis. The remaining patients are still at first clinical remission.

In conclusion, we have identified a minor BCR within the human *MLL* gene that is recurrently associated in acute leukemia patients with *MLL–USP2* fusion alleles as well as *MLL* fusion partnerships with *USP8*, *AF4*, and *AF9*. However, with 17 cases out of ~2500 analyzed patients the incidence is less than 1% while still ranking fourteenth of our updated fusion gene list (see Table 1 of reference 1). The discovery of a second, minor BCR extends our knowledge of the *MLL*-recombinome and *MLL*-r oncogenesis. Moreover, these findings will enable many labs to make changes in their diagnostic set-up for MLL-MRD diagnostics to ensure the best medical treatment for a group of patients that is still very hard to cure.

## Supplementary information

Supplementary data file
