# Supplementary material for: Human MLL/KMT2A gene exhibits a second breakpoint cluster region for recurrent MLL–USP2 fusions
Source: Leukemia. 2019 Mar 21;33(9):2306–40. doi: 10.1038/s41375-019-0451-7 (PMC6756029; doi:10.1038/s41375-019-0451-7)

Supplementary data file

Material and Methods

Patient material

Genomic DNA was isolated from bone marrow and/or peripheral blood samples of leukemia patients and sent to the DCAL (Frankfurt/Main, Germany). Patient samples were obtained from different study groups (the GMALL study group, Berlin; Polish Pediatric Leukemia and Lymphoma Study Group; Zabrze; I-BFM network) and diagnostic centers in and outside Europe where AL patients are enrolled in local study groups. Informed consent was obtained from all patients or patients' parents/legal guardians and control individuals.

Targeted sequencing approach

Customized oligonucleotide probes for the complete *MLL* gene were designed as capturing probes for DNA fragments of *MLL* gene after transposon-tagging. A total of 2,688 overlapping probes were designed using the DesignStudio (Illumina), including a panel of single nucleotide polymorphisms (SNPs) used as quality control for discrimination of patient samples.[1] For the establishment of the method, we used extracted DNA from the SEM cell line for targeted sequencing. By mixing SEM DNA with DNA of healthy cells, we were able to demonstrate that even 5% of blasts were sufficient to identify the corresponding chromosomal breakpoints. The Nextera DNA Library Prep Kit was used for library preparation, and subsequent paired-end sequencing was performed with MiSeq Reagent Kit v2/v3 (Illumina). Briefly, 50 to 65 ng genomic patient DNA were used for transposon-tagging, library preparation and subsequent DNA sequencing using an Illumina MiSeq platform. A uniform coverage distribution over the complete MLL gene was obtained with library fragments of 200-900 bp (mean 360 bp). This was important due of the many repetitive ALU elements present in the MLL gene, that would otherwise result in gaps when multimapping reads were excluded. Resulting data files (FASTQ files) were analyzed in our bioinformatic pipeline. Within the group of investigated patients, we had either limited (n=4) or no information (n=105) on their molecular status.

Data evaluation and statistical analyses

The structural variants (SV) breakpoint analysis was performed in several steps. As a first step, raw Illumina paired-end reads were processed with Trimmomatic v0.36[2] using the following parameters settings: ‘2:30:10 LEADING:3 TRAILING:3 SLIDINGWINDOW:4:15’. We screened routinely for a collection of Illumina adapter sequences obtained from https://support.illumina.com/downloads/illumina-adapter-sequences-document-

1000000002694.html. In step two, we identified known contaminants in Illumina data,[3] e.g. sequence reads stemming from genomic DNA of the bacteriophage phiX or from the bacterial genera *Propionibacterium*, *Roseateles*, *Bradyrhizobium*, *Geobacillus* and *Pelomonas*. To identify possible sample contaminations, we applied FastQ Screen v0.11.3[4] together with Bowtie2 v2.3.3.1[5] (--bowtie2 "--score-min G,10,8.4). In step three, we mapped single- and paired-end sequence reads against the human reference genome GRCh38.p10[6] with BWA mem v0.7.17-r1188[7] keeping the default parameter settings of BWA mem. Resulting SAM files were merged and sorted with SAMtools v1.9.[8] We then eliminated duplicates and marked split reads with SAMBLASTER v0.1.20[9] using the parameters '--excludeDups --addMateTags --maxSplitCount 2 --minNonOverlap 2'. To extract discordantly mapping read pairs and split reads, both hinting towards a SV, we used SAMtools together with the script "extractSplitReads_BwaMem" [script published in the LUMPY-SV source code repository [https://github.com/arq5x/lumpy-sv]. Precisely, the script was invoked as follows: 'samtools view -h -F 1294 sample.bam > sample.discordants.unsorted.bam && samtools view -h sample.bam | extractSplitReads_BwaMem -i stdin | samtools view -h -b > /mapping.svdetected/sample.splitters.unsorted.bam'. The resulting BAM file was sorted again, and we then used LUMPYexpress v0.2.13[10] to identify SVs, and SVTyper v0.6.0[11] for breakpoint genotyping these variants. The following program calls were used: 'lumpyexpress -B sample.sorted.bam -S sample.splitters.bam -D sample.discordants.bam -o sample.vcf && svtyper -B sample.sorted.bam -S sample.splitters.bam -i sample.vcf > sample.gt.vcf'.

Nomenclature

For the readability of the text, the following gene nomenclature was used throughout the text: *MLL* (*KMT2A*), *AF4* (*AFF1*), *LAF4* (*AFF3*), *AF5* (*AFF4*), *ENL* (*MLLT1*), *AF9 (MLLT3), AF6 (MLLT4), AF17 (MLLT6), AF10 (MLLT10), AF1Q (MLLT11)*. In addition, exon numbering is used according to the established *MLL* gene structure by our own group.[21]

Supplementary References

1. Pengelly RJ, Gibson J, Andreoletti G, Collins A, Mattocks CJ, Ennis S. A SNP profiling panel for sample tracking in whole-exome sequencing studies. Genome Med. 2013 Sep 27;5(9):89.
2. Bolger AM, Lohse M, Usadel B. Trimmomatic: a flexible trimmer for Illumina sequence data. Bioinformatics. 2014 Aug 1;30(15):2114-20.
3. Laurence M, Hatzis C, Brash DE. Common contaminants in next-generation sequencing that hinder discovery of low-abundance microbes. PLoS One. 2014 May 16;9(5):e97876.
4. Wingett SW, Andrews S. FastQ Screen: A tool for multi-genome mapping and quality control. Version 2. F1000Res. 2018 Aug 24 [revised 2018 Jan 1];7:1338.
5. Langmead B, Salzberg SL. Fast gapped-read alignment with Bowtie 2. Nat Methods. 2012 Mar 4;9(4):357-9.
6. Schneider VA, Graves-Lindsay T, Howe K, Bouk N, Chen HC, Kitts PA, Murphy TD, Pruitt KD, Thibaud-Nissen F, Albracht D, Fulton RS, Kremitzki M, Magrini V, Markovic C, McGrath S, Steinberg KM, Auger K, Chow W, Collins J, Harden G, Hubbard T, Pelan S, Simpson JT, Threadgold G, Torrance J, Wood JM, Clarke L, Koren S, Boitano M, Peluso P, Li H, Chin CS, Phillippy AM, Durbin R, Wilson RK, Flicek P, Eichler EE, Church DM. Evaluation of GRCh38 and de novo haploid genome assemblies demonstrates the enduring quality of the reference assembly. Genome Res. 2017 May;27(5):849-864.
7. Li H, Durbin R. Fast and accurate short read alignment with Burrows-Wheeler transform. Bioinformatics. 2009 Jul 15;25(14):1754-60.
8. Li H, Handsaker B, Wysoker A, Fennell T, Ruan J, Homer N, Marth G, Abecasis G, Durbin R; 1000 Genome Project Data Processing Subgroup. The Sequence Alignment/Map format and SAMtools. Bioinformatics. 2009 Aug 15;25(16):2078-9.
9. Faust GG, Hall IM. SAMBLASTER: fast duplicate marking and structural variant read extraction. Bioinformatics. 2014 Sep 1;30(17):2503-5.
10. Layer RM, Chiang C, Quinlan AR, Hall IM. LUMPY: a probabilistic framework for structural variant discovery. Genome Biol. 2014 Jun 26;15(6):R84.
11. Chiang C, Layer RM, Faust GG, Lindberg MR, Rose DB, Garrison EP, Marth GT, Quinlan AR, Hall IM. SpeedSeq: ultra-fast personal genome analysis and interpretation. Nat Methods. 2015 Oct;12(10):966-8.
12. Nilson I, Löchner K, Siegler G, Greil J, Beck JD, Fey GH, Marschalek R. Exon/intron structure of the human ALL-1 (MLL) gene involved in translocations to chromosomal region 11q23 and acute leukaemias. Br J Haematol. 1996;93: 966-72.

Supplementary Table S1A-C: Clinical and laboratory data of MLL-USP2 patients

All 3 Tables summarize the known data from the identified 17 MLL-USP2 cases, as well as the patients published by others. **A**. Clinical and laboratory data. **B**. FISH screening data. **C**. Clinical treatment and follow-up data.

Supplementary Figure S1: Scheme of MLL and USP2/8 proteins and their fusion sites. **A**. The protein domain structure is depicted, with red lines (above or below) indicating the exons structure of all three genes. Since both USP genes contain a first exon which is non-coding, both proteins start with exon 2. Major and minor BCR is depicted in the MLL protein structure, separated by the PHD1-3/BD domain. Breakpoints in the minor BRC are disrupting the PHD4 domain. Fusion of the UCH-domain to the extMLL is depicted. The major BCR fuses to 94 different partners. **B**. Part of the UCH domain is shown, displaying the CYC-box and the ASP-Box with the important histidine upstream. The catalytic mechanism has been unraveled in the paper from Zhang et al., in 2001 (the picture is from that paper). All 5 amino acids (position 271, 276, 557, 574 and 575 in the USP2 protein) important for deubiquitination are shown.

Supplementary Figure S2A-D: Summary of all identified breakpoint situations in 16 MLL-USP2 patients and the single MLL-USP8 case. A. Six reciprocal MLL-USP2 cases: **B**. 2 cases with reciprocal translocations, two cases with complex translocations; **C**. 3 cases with complex translocations; **D**: 5 cases with 3'-MLL deletions. In nearly all these cases larger deletions were accompanying the recombination event. The observed deletions were in the range of 10 bp up to 33,779 bp.

Supplementary Figure S3A-B: FISH analysis of MLL-USP2 fusions. Two main patterns were determined by FISH evaluation: **A.** The balanced inversion of ~870 Kb between MLL and USP2 creates direct and reciprocal fusions, which are visualized as a normal pattern by FISH evaluation. **B.** The inversion between MLL and USP2 is accompanied by deletion of MLL 3'. Therefore, the lack of one 3' probe (red) was observed.

Supplementary Table S1a-c

Supplementary Table S1a. Clinical and laboratory data of MLL-USP2 patients

ID	age at dx	gender	WBC (x10^9/L)	Leukemia	MLL-FP				FP-MLL			
					MLL intron	FP intron	Chrom	partner gene	FP intron	MLL intron	Chrom	partner gene
**1987**	10, yrs	m	3,4	B-ALL	Intron 22	Intron 2	11	USP2	Intron 1	Intron 31	11	C2CD2L
**2039**	,5 yrs	f	324,0	MPAL	Intron 21	Intron 2	11	USP2	-	-	-	Deletion
**3419**	1,6 yrs	f	16,2	B-ALL	Exon 22	Intron 2	11	USP2	-	-	-	Deletion
**3506**	2,1 yrs	f	NA	B-ALL	Intron 21	Intron 2	11	USP2	-	-	-	Deletion
**3576**	2,5 yrs	f	53,2	MPAL	Intron 16	Intron 2	11	USP2	Intron 2	Exon 37	11	USP2
**3586**	,9 yrs	m	9,1	MPAL	Intron 21	Intron 2	11	USP2	Intron 2	Intron 24	11	USP2
**3589**	,7 yrs	f	66,4	B-ALL	Intron 23	Intron 2	11	USP2	Intron 1	Intron 23	11	USP2
**3596**	3,5 yrs	m	8,0	B-ALL	Intron 23	Intron 2	11	USP2	18p11.32	Intron 24	18	18p11.32
**3613**	,3 yrs	f	41,6	MPAL	Intron 22	Intron 2	11	USP2	2p21	Intron 31	2	2p21
**3750**	,4 yrs	m	299,0	MPAL	Exon 23	Intron 2	11	USP2	Intron 1	Exon 23	12	WNT5B
**3787**	5,5 yrs	m	7,3	B-ALL	Intron 22	Intron 2	11	USP2	-	-	-	Deletion
**3798**	2,5 yrs	m	9,6	B-ALL	Exon 24	Intron 2	11	USP2	Intron 3	Intron 29	11	USP2-AS1
**3810**	2,7 yrs	m	126,6	B-ALL	Intron 23	Intron 2	11	USP2	Intron 2	Exon 23	11	USP2
**3811**	1,3 yrs	m	89,6	B-ALL	Intron 21	Intron 2	11	USP2	Intron 2	Exon 22	11	USP2
**3815**	1,2 yrs	f	68,0	B-ALL	Intron 21	Intron 2	11	USP2	-	-	-	Deletion
**3829**	2,4 yrs	f	7,6	B-ALL	Intron 21	Intron 2	11	USP2	Intron 2	Intron 21	11	USP2
**3875**	5,5 yrs	f	19,1	AML	Intron 21	Intron 2	11	USP2	-	-	-	Deletion
**SJBALL021549_D1**[$]	NA	f	NA	B-ALL	Intron 23	Intron 2	11	USP2	NA	NA	NA	NA
**SJINF066_D***	0,9 yrs	m	NA	B-ALL	Intron 23	Intron 2	11	USP2	NA	NA	NA	NA
**SJMPAL040026**[#]	0,5 yrs	m	355,5	MPAL	Intron 23	Intron 2	11	USP2	NA	NA	NA	NA
**SJMPAL043770**[#]	7,4 yrs	m	62,0	MPAL	Intron 21	Intron 2	11	USP2	NA	NA	NA	NA

Supplementary Table S1b. FISH screening data of MLL-USP2 patients

ID	FISH (ISCN 2016)	FISH interpretation	FISH image	Karyotype
**1987**	nuc ish(5'MLLx2,3'MLLx1)(5'MLL con 3'MLLx1)	MLL 3' deletion	No	48,XY,dup(1)(q21q31),add(4)(q?28),+8,+10,add(11)(q?23)[2]/46,XY[18]
**2039**	nuc ish(5'MLLx2,3'MLLx1)(5'MLL con 3'MLLx1)	MLL 3' deletion	No	NA
**3419**	nuc ish(5'MLLx2,3'MLLx1)(5'MLL con 3'MLLx1)	MLL 3' deletion	No	47,XX,+6[3]/47,sl,der(6)t(6,8)(q13;q13)[3]/47,sl,der(6)t(1,6)(q21;q13),der(17)t(8;17)(q13;p13)[14]/46,XX[3]
**3506**	nuc ish(5'MLLx2,3'MLLx1)(5'MLL con 3'MLLx1)	MLL 3' deletion	Yes	NA
**3576**	nuc ish(5'MLL,3'MLL)x1(5'MLL con 3'MLLx1)	MLL monoallelic loss	Yes	46,XX,del(11)(q23),der(16)t(1;16)(q11;q11)
**3586**	nuc ish(5'MLL,3'MLL)x2(5'MLL con 3'MLLx2)[196/200]	No MLL fusion	Yes	NA
**3589**	nuc ish(5'MLL,3'MLL)x2(5'MLL con 3'MLLx2)	No MLL fusion	Yes	NA
**3596**	nuc ish(MLLx2)(5 MLL sep 3 MLLx1)	MLL-r	Yes	NA
**3613**	nuc ish(5'MLL,3'MLL)x2(5'MLL con 3'MLLx2)	No MLL fusion	Yes	46,XX,t(2;11)(p21;q23)[17]/46,XX[3]
**3750**	nuc ish(5'MLL,3'MLL)x2(5'MLL con 3'MLLx2)	No MLL fusion	No	NA
**3787**	nuc ish(5'MLLx2,3'MLLx1)(5'MLL con 3'MLLx1)	MLL 3' deletion	No	NA
**3798**	nuc ish(MLLx2)(5'MLL sep 3'MLLx1)[23/121]	MLL-r	No	47,XY,+8[5]/46,XY[7]
**3810**	NA	NA	No	NA
**3811**	nuc ish(5'MLL,3'MLL)x2(5'MLL con 3'MLLx2)	No MLL fusion	No	NA
**3815**	nuc ish(5'MLLx2,3'MLLx1)(5'MLL con 3'MLLx1)	MLL 3' deletion	No	NA
**3829**	nuc ish(5'MLL,3'MLL)x2(5'MLL con 3'MLLx2)[99/100]	No MLL fusion	Yes	46,XX[16]
**3875**	nuc ish(5'MLL,3'MLL)x2(5'MLL con 3'MLLx2)	No MLL fusion	Yes	NA
**SJBALL021549_D1**[$]	NA	NA	No	NA
**SJINF066_D***	NA	NA	No	47,XY,+8[13]/46,XY[4]
**SJMPAL040026**[#]	NA	NA	No	46,XY,t(14;15)(q32;q11.2-13)[4/20], 46,XY[16/20]
**SJMPAL043770**[#]	NA	NA	No	NA

Supplementary Table S1c. Clinical treatment and follow-up data of MLL-USP2 patients

ID	Clinical trial	Risk group	CNS disease	MRD day 33	MRD day 78	PR	Relapse	Outcome	Follow-up
**1987**	FRALLE 2000	HR (B2)	Yes (CNS 2)	Negative	Negative	Good	No	1st CR	5,2 yrs
**2039**	ELAM02/Interfant-06	HR	Yes (CNS 2)	Positive	Positive	Poor	No	Dead (HSCT toxicity)	0,8 yrs
**3419**	Interfant-06 (NOS)	HR	NA	Negative	Negative	NA	NA	Dead	0,4 yrs
**3506**	CAALL_F01	HR	No	Positive	Negative	Good	No	1st CR	1,2 yrs
**3576**	Interfant	NA	Yes (CNS 2A)	Negative	Negative	NA	No	1st CR	5,0 yrs
**3586**	Interfant-06/AIEOP-BFM 2009	SR/HR	No	Positive	Negative	Good	No	1st CR (after HSCT)	5,0 yrs
**3589**	Interfant-06/FRALLE 2000 B2	HR	Yes (CNS 3)	Positive	Positive	Poor	No	1st CR (after HSCT)	1,4 yrs
**3596**	NOPHO ALL 2008	HR	No	Positive	Negative	NA	No	1st CR	1,2 yrs
**3613**	MLL-Baby	HR	No	Positive	Positive	Good	No	1st CR (after HSCT)	1,1 yrs
**3750**	Interfant-06	LR	No	Positive	Positive	Poor	No	1st CR (after HSCT)	4,2 yrs
**3787**	FRALLE 2000/personalized treatment	HR (B2)	NA	Positive	Negative	Good	No	1st CR (after HSCT)	1,0 yrs
**3798**	ANZCHOG ALL8 (NOS)	HR	NA	Positive	Positive	Good	No	1st CR	11,1 yrs
**3810**	AIEOP-BFM ALL 2000	HR	No	Positive	Positive	Poor	No	1st CR	6,2 yrs
**3811**	AIEOP-BFM ALL 2009	HR	No	Positive	Positive	Poor	No	1st CR	1,5 yrs
**3815**	CAALL_F01	HR	Yes (CNS 2)	NA	NA	Good	No	Induction ongoing	0,1 yrs
**3829**	CO-ALL 08/09	LR	No	Positive	Positive	NA	No	Consolidation ongoing	0,3 yrs
**3875**	AAML1031	HR	No	Positive	Positive	NA	BM	1st CR	0,2 yrs
**SJBALL021549_D1**[$]	NA	NA	NA	NA	NA	NA	NA	NA	0,4 yrs
**SJINF066_D***	NA	NA	NA	NA	NA	NA	NA	NA	NA
**SJMPAL040026**[#]	Japan (JACLS)	NA	NA	NA	NA	NA	No	Dead	0,1 yrs
**SJMPAL043770**[#]	AIEOP	NA	NA	NA	NA	NA	No	Alive	1,8 yrs

ALL: acute lymphoblastic leukemia; CNS: central nervous system disease; CR: complete remission; HR: high risk; HSCT: hematopoietic stem-cell transplantation; MPAL: mixed-phenotype acute leukemia; NA: not available; PT: personalized treatment; SR: standard risk; WBC: white blood cell count.

[$] Roberts et al. Targetable kinase-activating lesions in Ph-like acute lymphoblastic leukemia. N Engl J Med 2014;371:1005-1015.
* Andersson et al. The landscape of somatic mutations in Infant MLL rearranged acute lymphoblastic leukemias. Nat Genet 2015; 47:330–337.
[#] Alexander TB, Mullighan CG. The genetic basis and cell of origin of mixed phenotype acute leukaemia. Nature 2018; 562:373-379.

Supplementary Figures S1

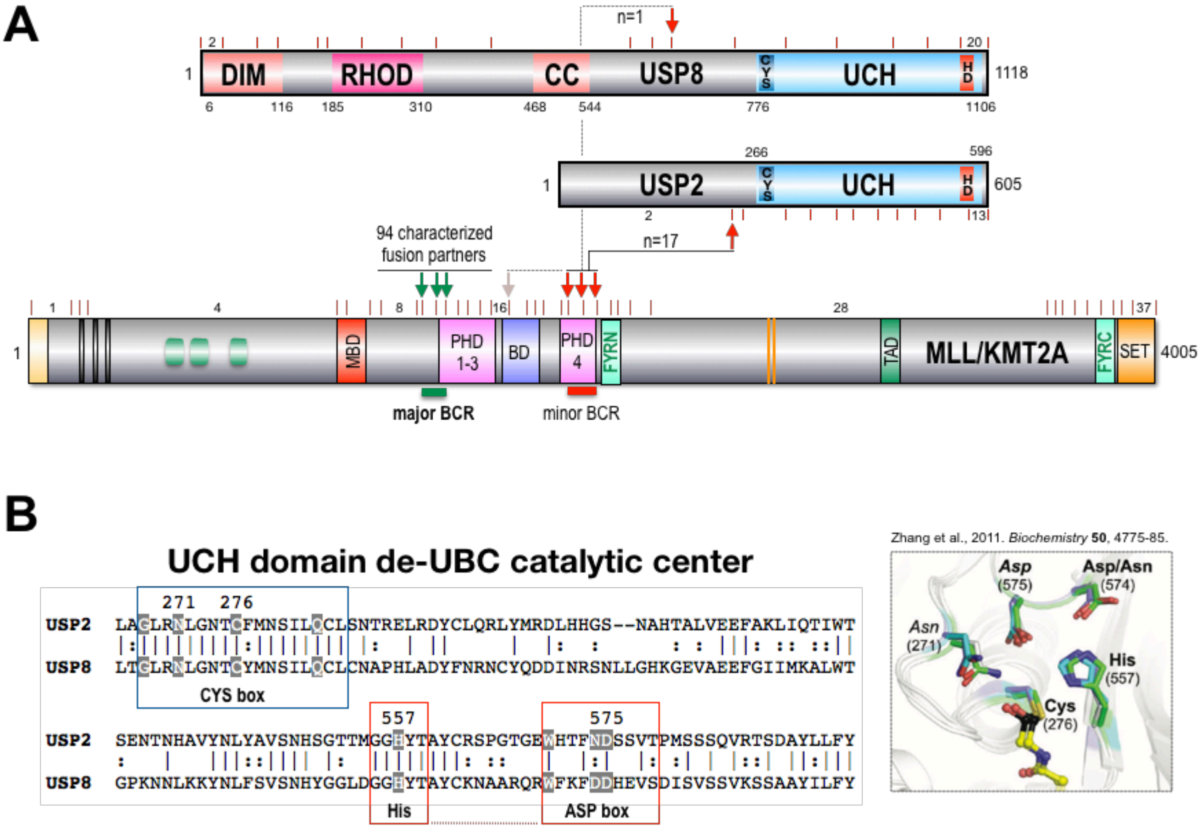

Supplementary Figures S2A

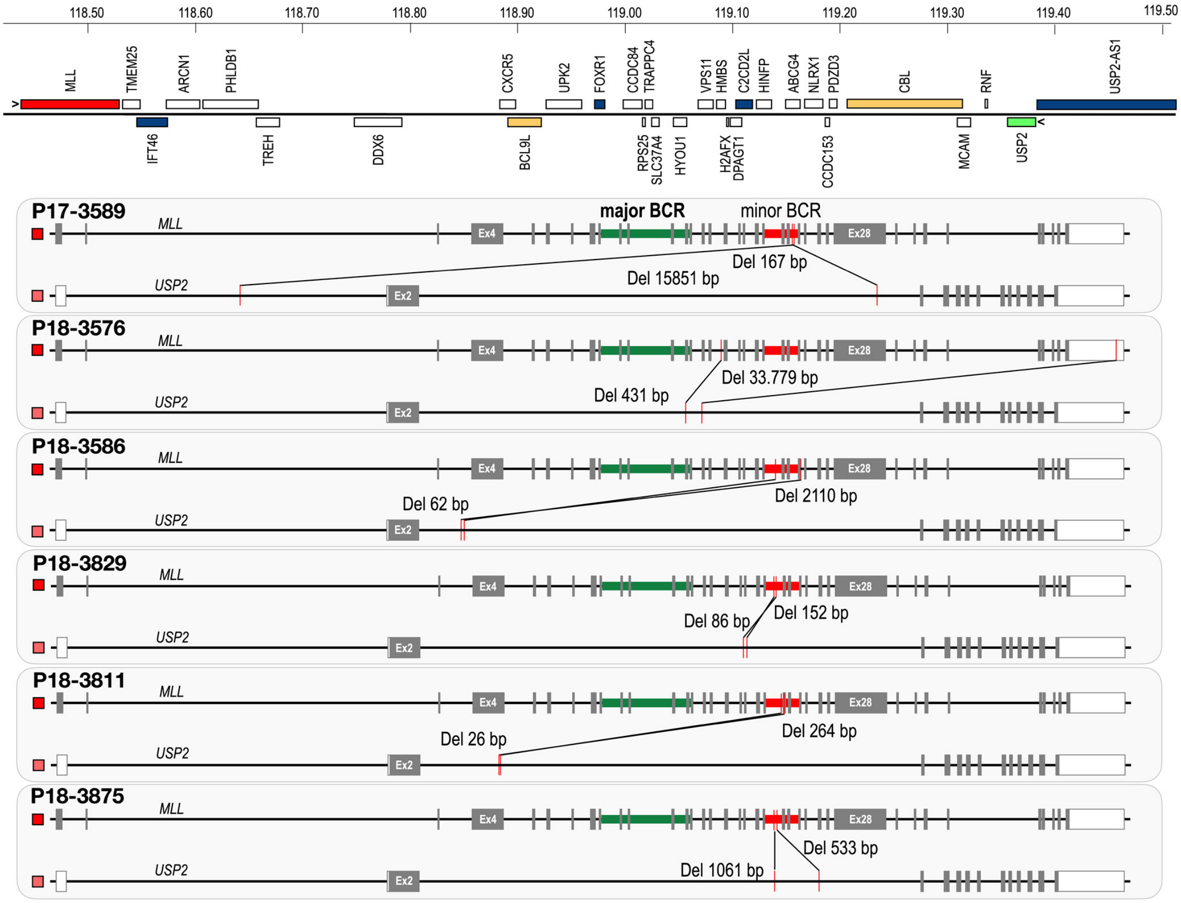

Supplementary Figures S2B

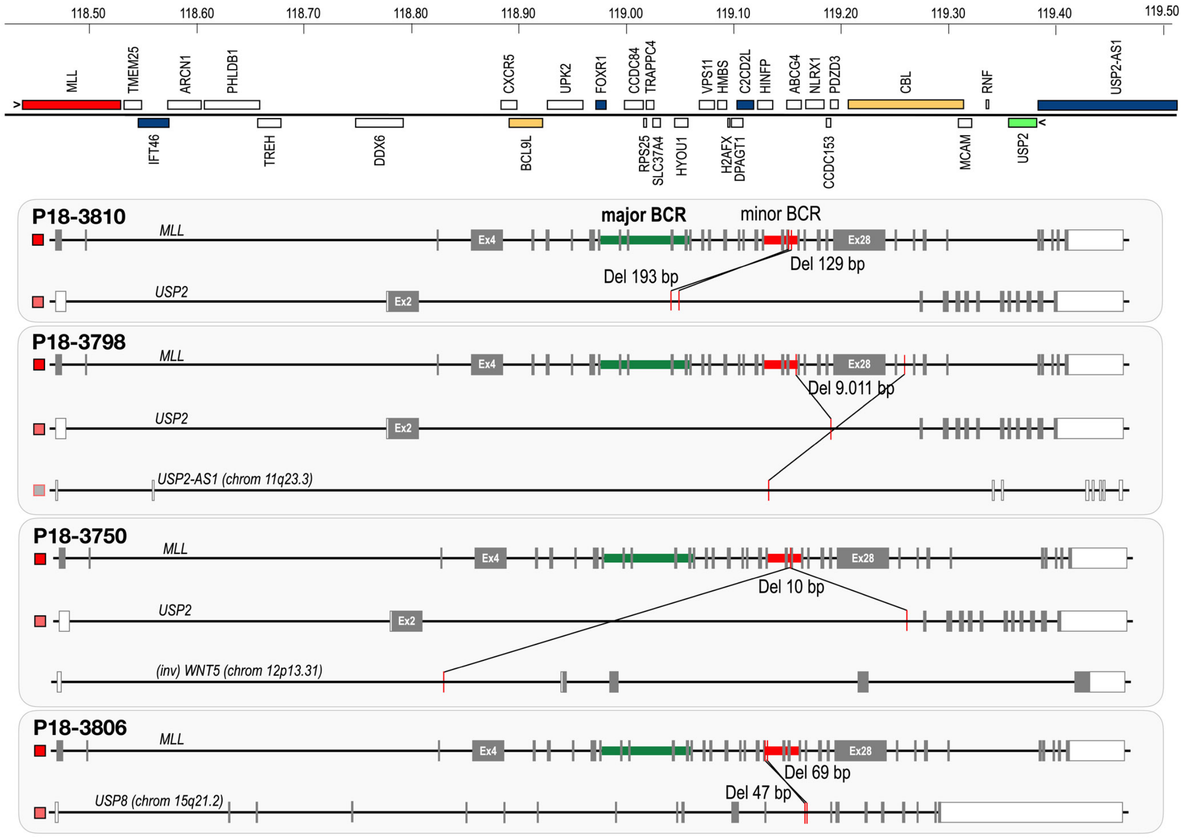

Supplementary Figures S2C

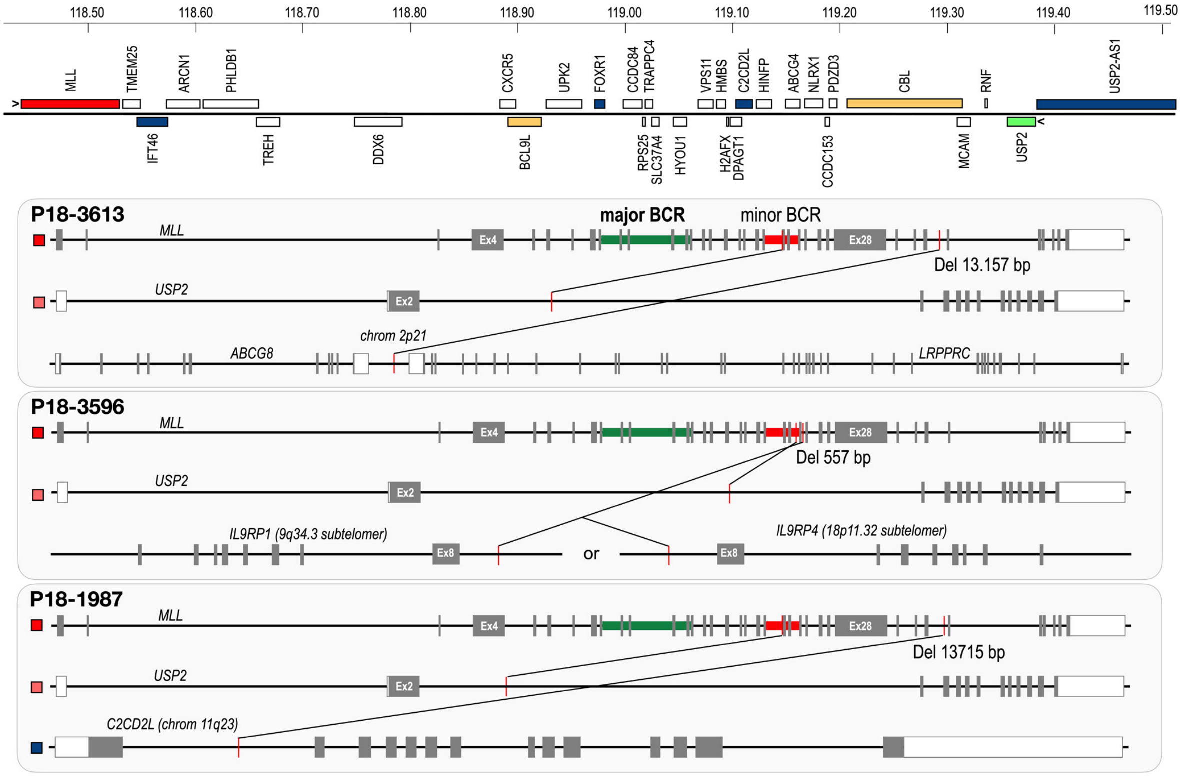

Supplementary Figures S2D

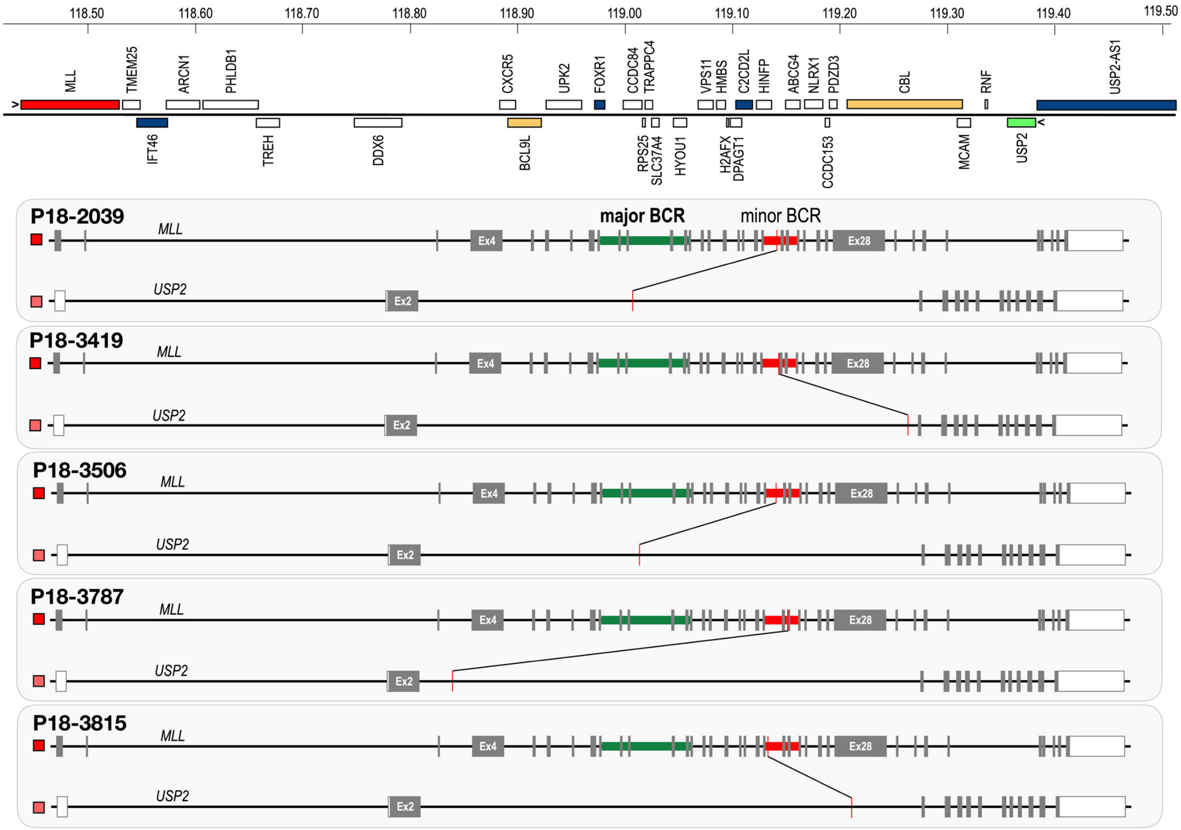

Supplementary Figures S3

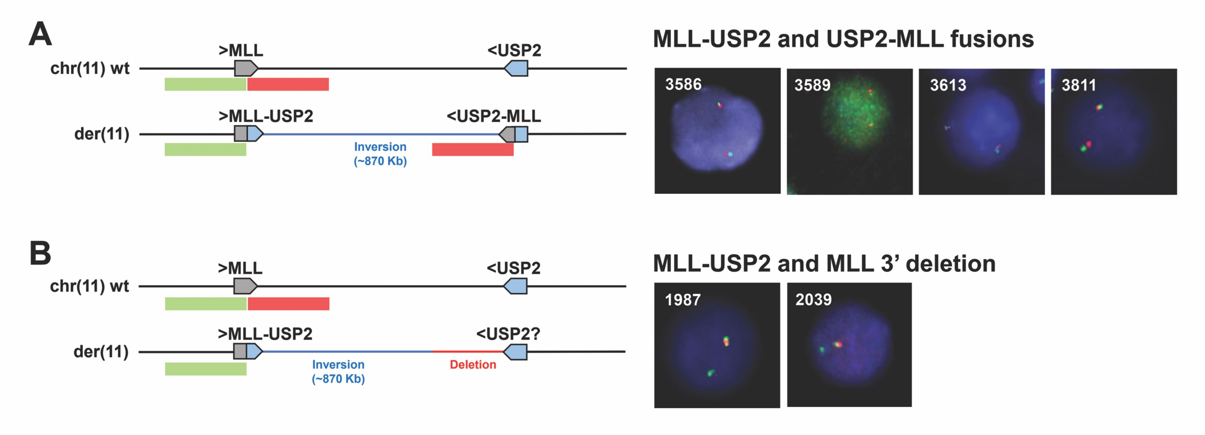

Supplement: Supplementary file 1 — Supplementary data file [file 41375_2019_451_MOESM1_ESM.pdf]
